# Differential diagnosis of systemic lupus erythematosus and rheumatoid arthritis with complements C3 and C4 and C-reactive protein

**DOI:** 10.3892/etm.2013.1304

**Published:** 2013-09-17

**Authors:** WENHUI LI, HUI LI, WUQI SONG, YUNLONG HU, YANHONG LIU, RONG DA, XIAOBEI CHEN, YANG LI, HONG LING, ZHAOHUA ZHONG, FENGMIN ZHANG

**Affiliations:** 1 Department of Microbiology, Harbin Medical University, Heilongjiang Key Laboratory of Infection and Immunity and Pathogenic Biology, Harbin, Heilongjiang 150081;; 2 Department of Laboratory Medicine, The Third Affiliated Hospital of Harbin Medical University, Harbin, Heilongjiang 150040;; 3 Departments of Laboratory Medicine, The Second Affiliated Hospital of Harbin Medical University, Harbin, Heilongjiang 150086, P.R. China; 4 Rheumatology, The Second Affiliated Hospital of Harbin Medical University, Harbin, Heilongjiang 150086, P.R. China

**Keywords:** complement C3, complement C4, C-reactive protein, systemic lupus erythematosus, rheumatoid arthritis, disease activity, differential diagnosis

## Abstract

The aim of this study was to analyze the changes in complements C3 and C4 and C-reactive protein (CRP) in patients with systemic lupus erythematosus (SLE) and rheumatoid arthritis (RA), and to evaluate the role of these indices in the differential diagnosis of SLE and RA. The first 347 patients with SLE, 382 patients with RA and 66 patients with erythema nodosum were selected for the measurement of complement and CRP levels in the serum, the erythema nodosum patients were the control group. The roles of the complements and CRP in the differential diagnosis and disease activity evaluation of SLE and RA were analyzed with SPSS 13.0. Complement C3 and C4 levels were significantly reduced in patients with SLE compared with those in the control group. However, in RA patients, the CRP level was increased. In addition, the levels of complements C3 and C4 in patients with SLE were much lower than those in patients with RA and the level of CRP in RA patients was much higher than that in patients with SLE. The reduction of complement C3 levels in SLE patients, and increase of CRP and complement C4 in patients with RA were associated with a higher risk of joint pain, butterfly rash and oral ulcer. These results show that the disease activity of SLE was negatively correlated with complement C3 and C4, and the disease activity of RA was positively correlated with CRP. With the increase in disease activity, the levels of complements C3 and C4 in patients with SLE were gradually reduced and the level of CRP in patients with RA was increased. There were distinctive differences in the levels of complements C3 and C4 and CRP between SLE and RA patients. The differences are useful in disease activity evaluation and the differential diagnosis of the two diseases that have similar symptoms.

## Introduction

Systemic lupus erythematosus (SLE) and rheumatoid arthritis (RA) are systemic autoimmune diseases that may attack the body’s cells and tissues, resulting in inflammation and tissue damage. The development processes and mechanisms of SLE and RA are not well established. Due to the similarity of symptoms (1,2), the differential diagnosis and treatment of these two diseases is challenging. Clinically, laboratory tests may be performed using various serum markers (3,4).

Certain differences in the pathogenesis, clinical symptoms and autoantibody changes between SLE and RA have been observed (5). These indicate that serum markers are able to reflect the differences between the two diseases in a wide range of aspects. In the current study, in order to illustrate the role of serum markers in the differential diagnosis and evaluation of the disease activity of SLE and RA, and to elaborate the different pathogenic mechanisms, we studied the roles of complements C3 and C4 and CRP, which are closely associated with the inflammatory response.

## Materials and methods

### Patient population

A total of 347 SLE patients and 382 RA patients were randomly selected from the individuals treated in The Second Affiliated Hospital of Harbin Medical University (Harbin, China) between 2009 and 2012. All patients provided informed consent and conformed with the American College of Rheumatology 1990 criteria for the classification of systemic lupus erythematosus (6,7). The study was approved by the Institutional Research Board of Harbin Medical Universiy. Clinical data acquisition and the evaluation of diagnosis and disease activity were performed by the same physician. The control group comprised 66 erythema nodosum patients.

### Disease activity and course evaluation

The assessments of SLE disease activity were performed with the Systemic Lupus Erythematosus Disease Activity Index (SLEDAI) rating system. According to the criteria of Gladman *et al* (8), the ratings were as follows: 0–9 for slight activity of SLE patients, 10–14 for moderate activity of SLE patients and ≥15 for severe activity of SLE patients. The assessments of RA disease activity were performed with the DAS28 rating system (9), which has the following ratings: <3.2 for slight activity, 3.2–5.1 for moderate activity and >5.1 for severe activity. On the basis of disease progression, fever and fatigue were determined as early symptoms, joint pain and butterfly rash were determined as metaphase symptoms and oral ulcers were determined as a late symptom.

### Serum samples

The serum samples were collected from the patients. C3, C4 and CRP were measured by latex-enhanced nephelometriy using high-sensitivity assays on the Behring analyzer (BN100 nephelometer; Dade Behring, Marburg, Germany). The concentration of CRP was normally below 5 mg/l. The normal ranges of complements C3 and C4 were 0.9–1.8 g/l and 0.1–0.4 g/l, respectively.

### Statistical analysis

Using SPSS 13.0 (SPSS, Inc., Chicago, IL, USA), the differences in the levels of complements C3 and C4 and CRP among the groups were compared with a Student’s t-test. The correlation of disease activity with C3, C4 and CRP was analyzed by linear correlation analysis. The data are presented as the mean ± SD. P<0.05 was considered to indicate a statistically significant result.

## Results

### Changes in C3, C4 and CRP in SLE and RA patients

**Figure 1.**
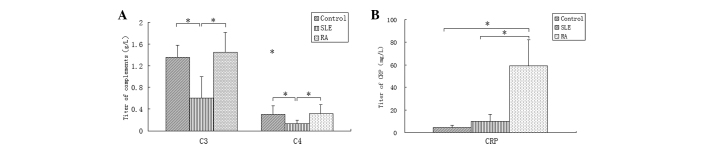
Titration and comparison of complements and CRP among control, SLE or RA patients. (A) The complement C3 and C4 titers of SLE patients (n=347) were significantly lower than those of RA patients (n=382) and controls (n=66; ^*^P<0.05). (B) The CRP titer of RA patients (n=382) was significantly higher than those of SLE patients (n=347) and controls (n=66; ^*^P<0.05). CRP, C-reactive protein; SLE, systemic lupus erythematosus; RA, rheumatoid arthritis.

From the clinical data and titers of serum markers, we observed that the levels of complements C3 and C4 in patients with SLE were lower than those of normal individuals and patients with RA (P<0.05; Fig. 1A). The CRP level in the patients with RA was higher than those of the control group and SLE patients (P<0.05; Fig. 1B).

### Differential diagnosis of SLE and RA with C3, C4 and CRP

The clinical data of controls, SLE and RA patients were collected, including gender, age, disease duration, DAS28 rating, SLEDAI rating, complement C3, complement C4 and CRP (Table I). Comparisons were performed between SLE and RA patients with similar symptoms. The results showed that in SLE and RA patients with fever and fatigue, there were no significant differences in the levels of complements C3 and C4 and CRP (P>0.05). However, in SLE patients with joint pain, butterfly rash and oral ulcer, complement C3 and C4 levels were markedly reduced (P<0.05). In RA patients with these symptoms, there was a significant increase of CRP (P<0.05; Table II). This suggests that when joint pain, butterfly rash and oral ulcer appear, patients with reductions in complement C3 and C4 levels and unchanged CRP levels may be diagnosed with SLE. By contrast, patients with an increased level of CRP and no changes in complement C3 and C4 levels may be diagnosed with RA. This suggests that although complements C3 and C4 and CRP did not show the differential diagnosis of SLE and RA with fever and fatigue, they may be useful for the differential diagnosis of SLE and RA with joint pain, butterfly rash and oral ulcer.

### Correlation of disease activity with C3, C4 and CRP

**Figure 2.**
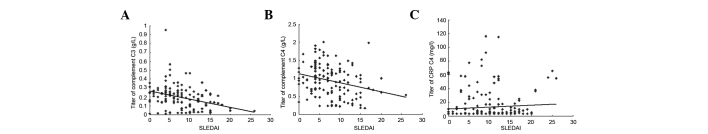
Relative analysis of the changes in C3, C4 and CRP with SLEDAI in SLE patients. The number of SLE patients was 347. Each point represents one pair of data (x and y value). There were significant negative and linear associations of (A) complement C3 (P<0.05, r=−0.535) and (B) complement C4 titers (P<0.05, r=−0.397) with SLEDAI. (C) No significant positive and linear association of CRP titer with SLEDAI rating was observed (P>0.05, r=0.068). CRP, C-reactive protein; SLE, systemic lupus erythematosus; RA, rheumatoid arthritis; SLEDAI, systemic lupus erythematosus disease activity index.

**Figure 3.**
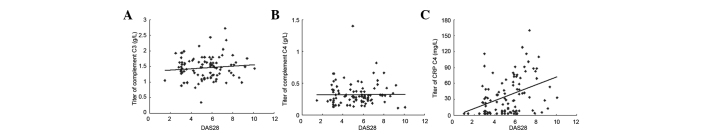
Relative analysis of changes in complements C3 and C4 and CRP with DAS28 scores in RA patients. The number of RA patients was 382. Each point represents one pair of data (x and y value). There was no significant positive and linear association of (A) complement C3 or (B) C4 titer with DAS28 score (P>0.05, r=0.014; P>0.05, r=0.099, respectively). (C) A significant positive and linear association of CRP titer with DAS28 score (P<0.05, r=0.386) is shown. CRP, C-reactive protein; SLE, systemic lupus erythematosus; RA, rheumatoid arthritis.

The SLE and RA patients were grouped as slight, moderate and severe according to the disease activity. The correlations of disease activity and changes in serum markers were analyzed. The results showed that complement C3 and C4 titers were negatively correlated with SLE disease activity (correlation coefficient r=−0.535 and −0.397 for C3 and C4, respectively, P<0.05; Fig. 2A and B). No significant correlation was identified between CRP titer and SLE disease activity (r=0.068, P>0.05; Fig. 2C). However, the CRP titer was positively correlated with the disease activity of RA (r=0.386, P<0.05; Fig. 3C). Complement C3 and C4 titers were not observed to be correlated with RA disease activity (r=0.014 and 0.099 for C3 and C4, respectively, P>0.05; Fig. 3A and B). As the disease activity of SLE and RA increased, complement C3 and C4 levels decreased (P<0.05) and the CRP level increased, respectively (P<0.05; Table III).

### Clinical prediction of SLE and RA with C3, C4 and CRP

Changes in serum markers were analyzed in SLE and RA patients according to the symptoms of different stages (Table IV). A higher risk of joint pain, butterfly rash and oral ulcer in SLE patients was demonstrated along with the reduction of complement C3, but not C4 (P<0.05). The upregulation of complement C4 and CRP increased the risk of joint pain, butterfly rash and oral ulcer in RA patients (P<0.05). However, in SLE patients, C4 and CRP levels did not significantly change during the course of the disease. These results suggest that complement C3 downregulation was a sign of the middle and late stages of SLE whereas complement C4 and CRP upregulation indicated the middle and late stages of RA.

## Discussion

Lupus and rheumatoid arthritis are typical autoimmune disorders. Since 80% of SLE and RA patients have fever and fatigue during the early stages, it is difficult to distinguish between these two disorders (10). Previous studies (11,12) have shown that blood markers may be used as an auxiliary diagnosis of the two diseases. However, the markers are nonspecific and their roles in the differential diagnosis, prediction and disease activity evaluation of SLE and RA are not well known. In addition, the role of serum markers in different regions and ethnicity has rarely been reported (13). In the present study, the role of complements and CRP in the differential diagnosis and evaluation of the disease activity of SLE and RA was analyzed.

The results showed that in the early stage of SLE and RA there were no clear differences in the titers of C3, C4 or CRP. However, in the middle and late stages, complement C3 and C4 levels were significantly reduced in SLE patients and CRP levels were increased in RA patients. This suggests that complements C3 and C4 and CRP are of no significance in the differential diagnosis of SLE and RA in the early stages. This may be associated with the slight inflammation at the beginning of these diseases. However, in the middle and late stages, there were significant reductions of complement C3 and C4 levels, but not of the CRP level in SLE patients. By contrast, in RA patients the CRP level was markedly reduced and no changes were observed in complement C3 and C4 levels. The different pathogenesis of SLE and RA may be due to the differences in serum markers, although inflammation is a common complication of SLE and RA. For example, in SLE patients, reduction of complement levels may be induced by the increase of kidney discharge and immune complex deposition while in RA patients, the CRP level was gradually increased and positively associated with the inflammation. These changes were in accordance with previous findings (14).

A previous study showed that complements C3 and C4 were reduced in Caucasian individuals with the disease activity of RA (15). However, only complement C4 was associated with SLE in individuals from Taiwan (Republic of China) (16). In the present study, we observed that in Northern China, complement C3 and C4 levels were decreased with SLE disease activity, whereas the level of CRP was not significantly changed. In patients with RA, the complement C3 and C4 levels tended to increase as the disease activity increased, but no significant differences were observed. Although a study of Caucasian SLE patients showed that CRP levels increased along with the SLEDAI scores (17), in the present study, no changes in the CRP titer were detected in SLE patients, although an increase was observed in RA patients. Our results show that the assessments of SLE and RA activity and progress in Northern China may be facilitated by the measurement of complements C3 and C4 and CRP. In addition, these data suggest that ethnicity affects the roles of C3, C4 and CRP in the differential diagnosis of SLE and RA.

The majority of patients with SLE and RA display fever and fatigue symptoms during the early stages of the disease. With the progress of the disease, patients present with joint pain and butterfly rash. Finally, microcirculation disorders and oral ulcers appear (18). On the basis of the disease process, we characterized fever and fatigue as early symptoms, joint pain and butterfly rash as metaphase symptoms and oral ulcer as late phase symptoms. This classification method was in accordance with the clinical symptoms of the majority of patients. Since this method of classification may reflect the dynamics of the disease, we applied it to assess the role of serum markers in the prediction and evaluation of the disease. In a previous study, complement C3 and C4 levels were observed to be reduced in SLE patients with joint pain, butterfly rash and oral ulcers. However, no marked changes in CRP levels were detected during this stage (10). In RA patients, the classical pathway of the complement system plays an important role in inflammation; this pathway may be activated by CRP (19). In a study in which RA patients were treated with anti-TNF-α antibodies, complement system levels were not reduced with CRP in certain patients. This suggests that the activation of complement system is not solely regulated by CRP (20). In our study, reduced complement C3 levels were significantly correlated with the middle and late stage symptoms of SLE, but not RA. Compared with the early stage, the increase of complement C4 and CRP was correlated with the middle and late stage symptoms of RA, but not SLE. The increase of complement C4 and CRP levels was correlated with the middle and late stage symptoms of RA, but not SLE. These findings are different from those of previous studies. The disease progression and genetic background of patients may have been different. Our study showed that C3, C4 and CRP are important for the differential diagnosis and prediction of clinical symptoms of SLE and RA.

Although there are certain differences in the roles of complements C3 and C4 and CRP in the differential diagnosis of SLE and RA (21,22), our results revealed that in SLE and RA patients of Northern China, C3, C4 and CRP may be used for the differential diagnosis, symptom prediction and disease progress evaluation. In addition, these results further demonstrated that athe reduction of complement should be caused by immune complex deposits, which contributed to the pathogenesis of SLE. By contrast, RA was mainly caused by inflammatory factors secreted from T cells following autoantigen changes. These results indicate that different inflammatory pathways may mediate the pathogenesis and development of SLE and RA. Although there were certain similarities in the symptoms, disease course and serum markers of SLE and RA, the changes in the inflammatory factors were detected to be heterogeneous. The nature of the disease differs between SLE and RA.

## Figures and Tables

**Table I.** Clinical data of the control, SLE and RA patients.

Clinical data	Controls (n=66)	SLE patients (n=347)	RA patients (n=382)
Male/female	9/57	26/321	42/340
Age (years)	43.72±14.61	35.15±11.49	47.66±12.48
Disease duration (months)	-	55.18±27.09	97.31±68.57
DAS28 rating	-	-	6.20±4.69
SLEDAI rating	-	9.23±5.14	-
Complement C3 (g/l)	1.36±0.22	0.61±0.39	1.45±0.36
Complement C4 (g/l)	0.31±0.15	0.14±0.06	0.32±0.17
CRP (mg/l)	4.53±1.84	10.40±5.88	59.12±22.79

Measurement data are expressed as mean ± SD. CRP, C-reactive protein; SLE, systemic lupus erythematosus; RA, rheumatoid arthritis.

**Table II.** Use of C3, C4 and CRP for the differential diagnosis of SLE and RA.

Clinical symptoms	Patients	Serum markers		
		C3 (g/l)^b^	C4 (g/l)^c^	CRP (mg/l)^d^
Malaise	SLE, n=22	0.84±0.37	0.13±0.09	18.49±13.15
	RA, n=18	1.35±0.42	0.26±0.12	43.00±41.91
	P-value	0.243	0.096	0.125
Fever	SLE, n=30	1.09±0.34	0.18±0.12	23.55±18.86
	RA, n=4	1.06±0.54	0.24±0.15	41.92±31.83
	P-value	0.907	0.324	0.704
Joint pain	SLE, n=33	0.81±0.39	0.14±0.10	17.89±11.00
	RA, n=236	1.29±0.43	0.27±0.16	43.56±32.58
	P-value	<0.001^a^	<0.001^a^	<0.001^a^
Butterfly rash	SLE, n=68	0.69±0.37	0.11±0.10	24.26±12.58
	RA, n=8	1.36±0.69	0.28±0.14	38.79±28.33
	P-value	0.031^a^	0.011^a^	0.045^a^
Oral cavity ulcer	SLE, n=10	0.75±0.43	0.19±0.17	22.23±11.02
	RA, n=3	1.36±0.41	0.24±0.18	51.06±41.99
	P-value	0.022^a^	<0.001^a^	<0.001^a^

a P<0.05, significant difference between the SLE and RA patients;

b normal range 0.9–1.8 g/l;

c normal range 0.1–0.4 g/l;

d normal range≤5 mg/l. Measurement data are expressed as mean ± SD. CRP, C-reactive protein; SLE, systemic lupus erythematosus; RA, rheumatoid arthritis.

**Table III.** Evaluation of C3, C4 and CRP in SLE and RA disease activity.

Serum markers	Activity level in SLE patients			Activity level in RA patients		
	Slight (n=119)	Moderate (n=85)	High (n=28)	Slight (n=35)	Moderate (n=139)	High (n=208)
C3 (g/l)	1.00±0.39	0.59±0.32^a^	0.53±0.24^a^	1.38±0.38	1.43±0.34	1.48±0.37
C4 (g/l)	0.19±0.10	0.10±0.04^a^	0.08±0.06 ^a^	0.30±0.11	0.30±0.14	0.35±0.21
CRP (mg/l)	17.24±12.87	16.13±10.17	16.00±9.77	20.99±19.53	27.04±16.72	53.79±42.71^a,b^

Data presented as mean ± SD.

a P<0.05, significantly different from the low activity group;

b P<0.05, significantly different from the moderate activity group. CRP, C-reactive protein; SLE, systemic lupus erythematosus; RA, rheumatoid arthritis.

**Table IV.** Predictive value of C3, C4 and CRP for the disease progression of SLE and RA.

Serum markers	SLE patients			RA patients		
	Fatigue, fever (n=144)	Joint pain, butterfly rash (n=145)	Oral cavity ulcer (n=58)	Fatigue, fever (n=39)	Joint pain, butterfly rash (n=310)	Oral cavity ulcer (n=33)
C3 (g/l)	0.84±0.37	0.75±0.38^a^	0.67±0.35^a,b^	1.35±0.42	1.33±0.43	1.50±0.30
C4 (g/l)	0.13±0.09	0.13±0.11	0.15±0.11	0.26±0.12	0.28±0.16	0.33±0.17^a,b^
CRP (mg/l)	18.49±13.15	15.42±13.08	13.08±12.12	41.00±38.91	46.03±32.49^a^	49.83±33.05^a,b^

Data presented as mean ± SD.

a P<0.05, significant difference from early symptoms (fatigue and fever);

b P<0.05, significant difference from metaphase symptoms (joint pain and butterfly rash). CRP, C-reactive protein; SLE, systemic lupus erythematosus; RA, rheumatoid arthritis.
